# Histopathological study of lesions of the caruncle: a 15-year single center review

**DOI:** 10.1186/1746-1596-4-29

**Published:** 2009-08-31

**Authors:** Helena P Solari, M Palis Ventura, Maria Eugenia Orellana, Gustavo A Novais, Devinder P Cheema, Miguel N Burnier

**Affiliations:** 1 Henry C. Witelson Ocular Pathology Laboratory, McGill University, Montreal, Canada; 2 Fluminense Federal University, Rio de Janeiro, Brazil

## Abstract

**Introduction:**

The caruncle is a modified cutaneous tissue located at the inner canthus that contains hair follicles, accessory lacrimal glands, sweat glands and sebaceous glands. These different types of tissues can give rise to a wide variety of lesions that make the clinical diagnosis difficult. The aim of the study was to investigate the most common types of caruncle lesions and the clinical and pathological correlation.

**Methods:**

Retrospective, observational case series. Records of caruncle lesions examined at the Henry C. Witelson Ocular Pathology Laboratory, McGill University, Montreal, Canada, between 1993 and 2008 were analyzed, comparing the clinical and histopathological findings.

**Results:**

A total of 42 lesions from 42 patients were analyzed. Twenty-six (61.90%) of the patients were women and 16 (38.10%) were men and the age range from 20 to 84. The main diagnoses were: 16 epithelial lesions (38.09%), 14 inflammatory lesions (31.70%), 10 melanocytic lesions (21,95%), 2 lymphoid lesions (4.87%). From the 28 cases that had a preoperative clinical hypothesis only 17 presented a histopathological confirmation of the diagnosis (60.71%).

**Conclusion:**

The most common caruncle lesions were epithelial tumors followed by chronic inflammation and melanocytic lesions. Although most of the lesions were benign, there was a great number of misdiagnose based on the clinical suspicious.

## Background

The caruncle is a modified cutaneous tissue located at the inner canthus of the eye, medial to the plica semilunaris. It is attached to the medial rectus and contains hair follicles, accessory lacrimal glands, sweat glands, lobules of fat, and sebaceous glands [1-3]. These different types of tissues can give rise to a wide variety of lesions; most of them benign, but the variety of lesions that affect the caruncle make the clinical diagnosis difficult [3,4]. Caruncle lesions usually show inconsistency between clinical and histopathological diagnosis, which can be as high as 50% [3,5,6].

The aim of this study was to determine the frequency and describe the histopathological features of caruncle lesions over a 15-year period.

## Methods

A single-center, retrospective analysis was performed on 42 records of 42 caruncle lesions from The Henry C. Witelson Ophthalmic Pathology Laboratory and Registry, McGill University, Montreal, Canada, over a period of 15 years, comparing the clinical and histopathological findings.

## Results

A total of 42 lesions from 42 patients were analyzed. Twenty-six (61.90%) of the patients were women and 16 (38.10%) were men and the age range from 20 to 84. The main diagnoses were: 16 epithelial tumors (38.09%), 14 inflammatory lesions (33.33%), 10 melanocytic lesions (23.80%) and 2 lymphoid lesions (4.87%). The results included seventeen different histopathological types of lesions. (Table 1)

**Table 1:** Classification of lesions of the caruncle

**Histopathological diagnosis**	**n**	**%**	**mean age (y)**	**age range (y)**	**male**		**female**	
					**n**	**%**	**n**	**%**
**Epithelial Tumors**								
Epidermoid cyst/epithelial inclusion cyst	3	7.14%	46,66	34-68	1	33.33%	2	33.33%
Squamous cell papilloma	3	7.14%	38	30-52	1	33.33%	2	66.66%
Oncocytoma	3	7.14%	59.66	52-67	1	33.33%	2	66.66%
Eccrine cyst	2	4.76%	53.50	32-70		-	2	100%
Sebaceous adenoma	1	2.38%	51	-		-	1	100%
Conjunctival cyst	1	2.38%	84	-		-	1	100%
Intraepithelial dysplasia	1	2.38%	48	-		-	1	100%
Papilloma	1	2.38%	30	-		-	1	100%
Basal cell carcinoma	1	2.38%	58	-				
**Inflammatory Lesions**								
Chronic inflammation	12	29.28%	67.50	50-72	8	66.66%	4	33.33%
Inflammation and epithelial hyperplasia	1	2.38%	66	-		-	1	100%
Granulomatous inflammation	1	2.38%	76	-		-	1	100%
**Melanocytic Lesions**								
Subepithelial nevus	3	7.14%	63.33	50-78	1	33.33%	2	66.66%
Compound nevus	6	14.64%	43.16	20-85	2	33.33%	4	66.66%
Primary acquired melanosis with mild atypia	1	2.38%	38	-		-	1	100%
**Lymphoid Tumors**								
Lymphoid hyperplasia	1	2.38%	78	-		-	1	100%

Low-grade B cell lymphoma	1	2.38%	34	-		-	1	100%

**Figure 1:**
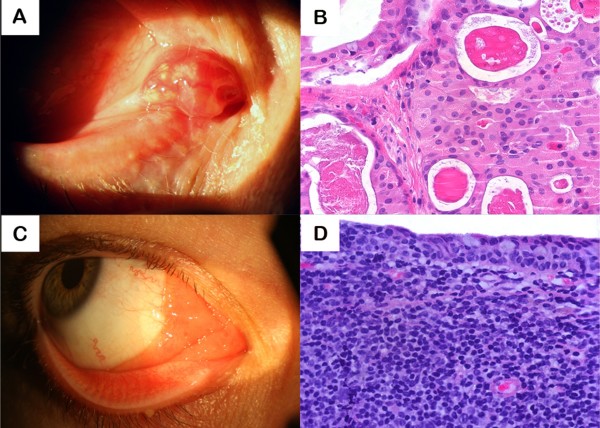
Oncocytoma and MALT Lymphoma.  Oncocytoma (A, B): **A**, clinical appearance and **B**, histopathology (HE 400×) showing sheets of large granular, eosinophilic polyhedral epithelial cells (oncocytes) having small, round, benign appearing central nuclei with large nucleoli. MALT Lymphoma (C, D): **C**, clinical appearance and **D**, histopathology showing lymphoid B cells infiltrating the caruncle's surface epithelium (HE 400×).

The most commonly observed lesions were the epithelial tumors that consisted of: epidermoid cyst/epithelial inclusion cyst (n = 3, 7.14%), squamous cell papilloma (n = 3, 7.14%), oncocytoma (n = 3, 7.14%), eccrine cyst (n = 2, 4.76%), sebaceous adenoma (n = 1, 2.38%), conjunctival cyst (n = 1, 2.38%), intraepithelial dysplasia (n = 1, 2.38%), papilloma (n = 1, 2.38%), and basal cell carcinoma (n = 1, 2.38%). (Figure 1)

Inflammatory lesions accounted for 33.33% of the cases, including: chronic inflammation (n = 12, 28.56%), inflammation and epithelial hyperplasia (n = 1, 2.38%) and granulomatous inflammation (foreign body) (n = 1, 2.38%).

The melanocytic lesions were detected in 23.80% of the cases and included: subepithelial nevus (n = 3, 7.14%), compound nevus (n = 6, 14.28%), one of which presented atypia, and a primary acquired melanosis with mild atypia (n = 1, 2.38%). The lymphoid tumors were: lymphoid hyperplasia (n = 1, 2.38%) and a low-grade B cell lymphoma MALT (n = 1, 2.38%). (Figure 1)

The samples included 40 benign lesions and only two malignant tumors, a basal cell carcinoma and a low-grade B cell lymphoma MALT. Two pre-malignant lesions were diagnosed, one case of intraepithelial dysplasia and one primary acquired melanosis with mild atypia. In the melanocytic group one of the nine nevi had atypia.

## Discussion

The conjunctiva and its associated elements, the caruncle and the plica semilunaris, form a smooth, flexible protective sac that covers the pericorneal surface of the anterior portion of the eye and lines the posterior surface of the eyelids [7]. The caruncle is a transition zone combining mucous membrane and adnexal elements. It is composed of a round-to-ovoid head and a tail blending with the skin at the canthus. The epithelial and subepithelial components of the caruncle reflect the partial derivation of this structure from the eyelid. The epithelium is thick and of the nonkeratinized stratified squamous type. The stroma contains fibroblasts and melanocytes interspersed with collagen, sebaceous glands, hair follicles, and striated muscle fibers derived from the orbicularis oculi muscle (Horner's muscle). In some individuals, serous glandular elements are found [1,7]. The caruncle is supplied by the superior medial palpebral artery, the lymphatics drain to the submandibulary lymph nodes and it is innervated by the intratrochlear nerve [8].

Most tumors that arise in the adnexal structures of the eyelids, eyebrows, and orbit may also originate in the caruncle [1,2,5,9,10]. The clinical differential diagnosis includes benign and malign tumors caused by melanocytic, epithelial, inflammatory, lymphocytic and vascular lesions [2,9-12]. As caruncle tumors have varied presentations it is commonly misdiagnosed. In most of the cases the clinical diagnosis is uncertain and the biopsy is indicated to confirm the diagnosis. Accordingly to literature our results demonstrated that most of the tumors are benign lesions.

In the head and neck, squamous cell carcinoma is by far the most common type of malignancy, and almost all malignant cellular changes are related to chronic irritation. Excluding the skin and thyroid gland, more than 90% of head and neck cancers are squamous cell carcinomas, and 5% are melanomas, lymphomas, and sarcomas. Most head and neck cancers can spread to the lymph nodes of the neck [13]. Differently from the head and neck tumors, most of the tumors of caruncle are benign. The malignant tumors are rare, and can also spread to submandibular lymph nodes [2,3,5].

In this study, the most common cause of caruncle lesions was epithelial tumors (38.09%) followed by inflammation (33.33%) and melanocytic tumors (23.80%). Considering all the seventeen different histopathological diagnosis in the study, compound nevus was the second most common cause of caruncle lesion.

Previous studies reported melanocytic lesions and papilloma as the most frequently excised lesions [2,5,11,14]. Luthra et al. [3] reported a review of 112 caruncular lesions during a 52-year period. The pigmented lesions were the most commonly encountered lesions accounting for 46.4% and epithelial lesions (15.17%) were the second most frequent. Kaeser et al. [2] described a series of 195 caruncle lesions, most of them from melanocytic origin (n = 96) including nevus, dysplastic nevus, primary acquired melanosis and malignant melanoma. The second most common group of lesions was caused by benign epithelial tumors, 37%, a percentage similar to our findings. The authors described only 3 malignant tumors, including: melanoma, sebaceous carcinoma and low-grade malignant lymphoma. In this paper the preoperative clinical diagnosis was confirmed by histopathology in 37.4% of the cases [2]. In a previous study, Ostergaard et al [15] reported 574 caruncular lesions, from a 25-year period, and 96% of the tumors were benign (nevus and papilloma). In this study the preoperative clinical diagnosis was correct in 50% of the cases, that corroborate our results [15]. Our reports demonstrated a lower incidence of melanocytic tumors than previous published data with a higher incidence of epithelial tumors, followed by inflammatory causes. These findings can be explained by the fact that inflammatory lesions presents hemossiderin pigment that can lead to a clinical misdiagnosis of melanocytic lesion, and also inflammatory lesions usually presents in older patients leading to clinical suspicion of malignancy that ends up in biopsy procedure [2,4,14]. The analysis of clinical data indicated that squamous cell papillomas affected youngest patients, with a mean age of 38 years, and most of the patients were female. On the contrary, inflammatory lesions were more frequent in males. Considering the melanocytic group, two of the ten tumors (20%) had atypia, one compound nevus and a primary acquired melanosis, indicating the importance of biopsy in suspicious lesions. The incidence of malignant lesions (4.76%) was similar to previous reports [2,3,14].

According to previous reports in our study only 17 from the 28 cases that had a preoperative clinical hypothesis presented a histopathological confirmation of the diagnosis (60.71%) [4,11,14].

## Conclusion

The most common caruncle lesions were caused by epithelial tumors followed by chronic inflammation and melanocytic lesions. The rarity and variety of caruncle lesions turn the clinical diagnosis difficult indicating the importance of biopsy to confirm the pre-operative hypothesis. Although most of the lesions were benign, there was a great number of misdiagnose based on the clinical suspicious. Referral to histopathological evaluation is mandatory to establish the best management.

## Competing interests

The authors declare that they have no competing interests.

## Authors' contributions

HPS participated in the design of the study, and in the histopathologic analysis. MPV and DPC performed the statistical analysis. MEO and GAN carried out the histopathologic studies. MNB Jr conceived of the study, and participated in its coordination. All authors read and approved the final manuscript.
